# Meta-narrative review: the impact of music therapy on sleep and future research directions

**DOI:** 10.3389/fneur.2024.1433592

**Published:** 2025-01-07

**Authors:** Qiaoqiao Gou, Meihui Li, Xiaoyu Wang, Xinran Yuan, Mingyi Yang, Junrui Li, Bo Wang, Dan Yang, Xiubo Ren, Miaomiao Yang, Siqi Liu, Ningning Liu, Jiaqi Han, Qiujian Xu

**Affiliations:** ^1^Art Healing and Cognitive Science Research Center, Department of Music, School of Arts and Design, Yanshan University, Qinhuangdao, China; ^2^YSU and DCU Joint Research Centre for the Arts, Music College, Daegu Catholic University, Daegu, Republic of Korea

**Keywords:** sleep, sleep disorders, music therapy, insomnia, meta-narrative review

## Abstract

Sleep is essential to human health, yet 27% of the global population suffers from sleep issues, which often lead to fatigue, depression, and impaired cognitive function. While pharmacological treatments exist, non-pharmacological approaches like music therapy have shown promise in enhancing sleep quality. This review, analyzing 27 studies with various experimental paradigms, confirms that music therapy significantly improves subjective sleep quality, largely by alleviating anxiety and regulating mood through perceptual pathways. However, the effects on objective sleep measures remain inconclusive, suggesting that individual differences may play a significant role. Future research should focus on refining intervention designs that integrate both subjective and objective sleep assessments to better elucidate the physiological and psychological mechanisms of music therapy. Key recommendations include personalized music selection, development of age-appropriate interventions, and minimization of external interferences to maximize therapeutic outcomes. Additionally, incorporating variables like psychological status, lifestyle, and environmental factors may offer a more comprehensive understanding of music therapy’s long-term adaptability and effectiveness for diverse populations. This review offers critical research directions and practical support for future applications of music therapy in sleep health.

## 1 Introduction

Good sleep is essential for maintaining optimal cognitive function, immune response, and overall health (1). However, according to a World Health Organization survey, 27% of the global population suffers from sleep disorders (2), with insomnia frequently causing significant negative impacts on individuals’ physical and mental well-being (3). Sleep disorders mainly involve issues related to sleep quality, sleep duration, and abnormal behaviors, including conditions such as insomnia, hypersomnia, and sleep apnea syndrome. Insomnia-related sleep deprivation is a primary factor contributing to suboptimal health (4). As one of the most prevalent clinical issues (5), sleep-related problems can further lead to other health concerns, such as impaired concentration and memory loss (6), and increase the risk of diabetes, cancer, cardiovascular diseases (7), and neurological disorders (8). Additionally, these issues indirectly interfere with daily tasks, making sleep problems one of the leading causes of traffic accidents (9). Furthermore, sleep problems are often comorbid with mental health issues such as depression (10) and anxiety (11), with a bidirectional relationship that can exacerbate existing conditions and hinder the efficacy of psychological interventions (12, 13).

This widespread health issue has drawn global researchers’ attention, prompting a range of potential solutions, among which music as a non-pharmacological intervention has garnered significant interest. Music therapy (MT), which are low-cost and free of side effects, have been demonstrated to improve sleep quality (14–17). MT’s calming effect on the parasympathetic nervous system can reduce anxiety, blood pressure, heart rate, and respiratory frequency (18), all of which are indicators commonly affected in sleep disorders. These findings support the hypothesis that MT can effectively alleviate sleep disturbances, reduce insomnia symptoms, facilitate sleep onset, and enhance sleep quality (19, 20). This discovery has rapidly captured worldwide researchers’ interest, with the type of music most effective for sleep disorders emerging as a key research focus. Sleep-promoting music may share common objective musical characteristics (21). Empirical studies focusing on intrinsic musical parameters, such as rhythm (22), timbre (23), pitch (24), and genre (25–27), have gained scholarly attention. For example, Ryu et al. found that sleep-inducing music with tempos of 60–80 beats per minute could improve sleep quality (28, 29). The second prominent research focus is on exploring experimental paradigms to verify the efficacy of MT, with neural signal detection emerging as a promising empirical model. Musical input directly affects brain wave patterns and peaks (30), and Electroencephalography (EEG) signals provide an observable means of identifying sleep stages (31), offering a feasible empirical paradigm for inducing, monitoring, and modulating sleep states in music-based conditions. The third frequently discussed topic is the impact of demographic factors on intervention effectiveness. Existing evidence indicates that intervention outcomes may vary by participants’ age or health conditions, indirectly suggesting potential variations in music intervention efficacy across individuals, age groups, and health conditions (32, 33).

It is evident that current research on MT’s impact on sleep disorders is extensive, diverse, and of significant value. However, these studies have yet to be systematically organized and synthesized. To offer a comprehensive understanding and insight into this field, this review aims to systematically consolidate and integrate empirical research on music’s effects on sleep health in recent years. By focusing on three widely discussed aspects—types of music, experimental paradigms, and demographic variables—this review provides a theoretical foundation for music’s therapeutic value in sleep disorders, guiding future empirical research and clinical practice toward more in-depth and comprehensive explorations.

## 2 Methods

### 2.1 Inclusion and exclusion criteria

This review employs the meta-narrative review approach, a method that integrates different types of research and methodologies, providing a multi-perspective research view. This approach offers valuable insights into understanding the research progress within a specific field, guiding future research directions, and supporting decision-making (34, 35). The primary aim of this review is to investigate the intervention potential of MT for sleep issues by exploring various sleep-related disorders and diverse populations. Additionally, we aim to provide readers with more detailed insights into the efficacy of MT by analyzing gaps in prior research and suggesting research designs and methodological considerations that may be better suited for future studies. Therefore, empirical studies that align with the research theme are included in this review (Table 1).

**Table 1:** Inclusion and exclusion criteria of studies.

Inclusion criteria	Exclusion criteria
Study subjects may include participants of all age groups, with or without sleep disorders.	All non-empirical research article types.
Studies must use at least one subjective measure of sleep or report the impact of music intervention on at least one of the following sleep parameters: Total Sleep Time (TST), Sleep Onset Latency (SOL), Sleep Efficiency (SE), Wake After Sleep Onset (WASO), Duration of Each Sleep Stage, Rapid Eye Movement (REM) Sleep Latency, Spectral Power of EEG During Sleep, EEG Arousal Index, Cyclic Alternating Pattern (CAP), etc.	Studies not using music as a variable or studies where music comprises a minimal component of combined therapies.
	Articles not in English or not published in peer-reviewed English-language journals.

### 2.2 Literature search strategy

**Figure 1:**
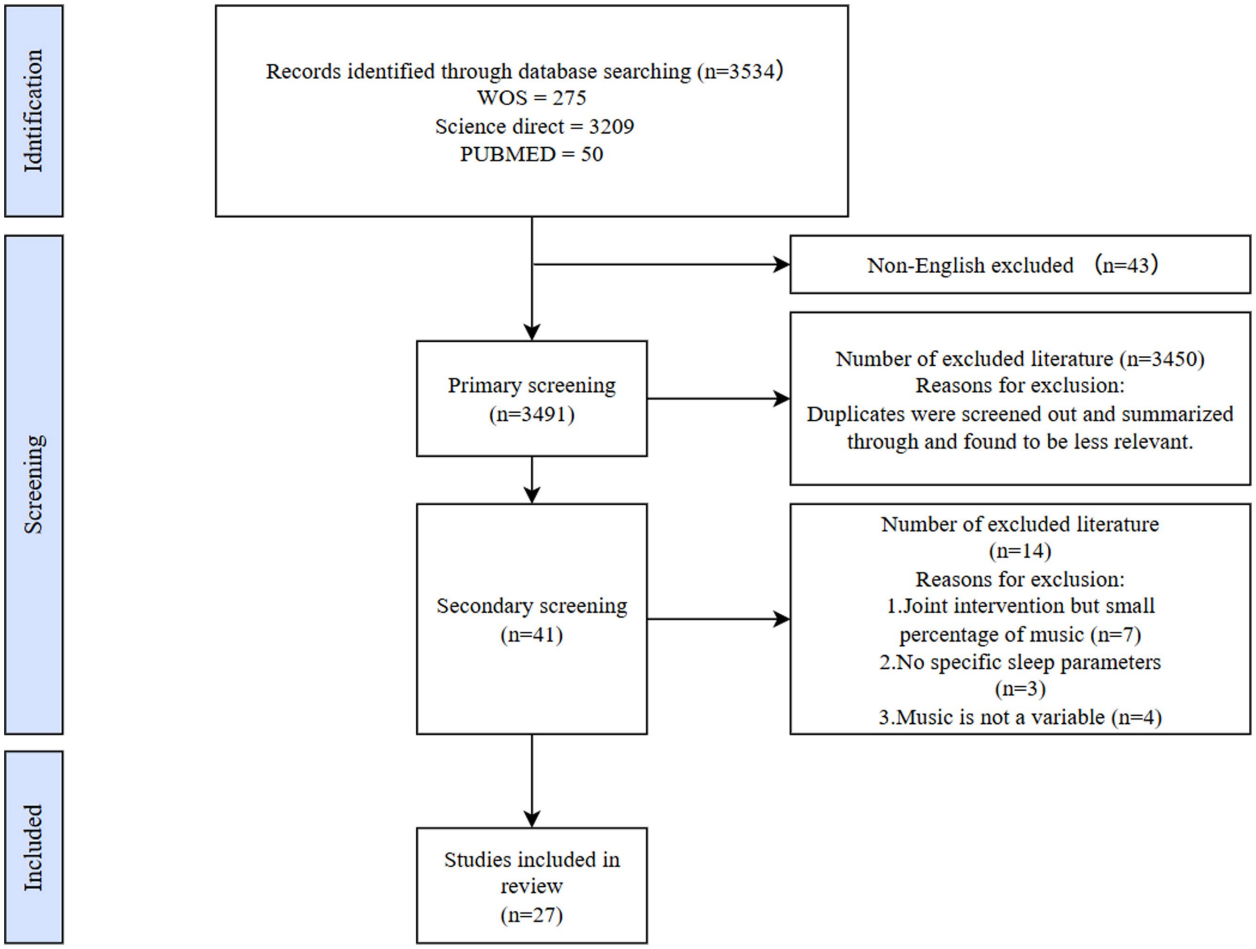
Flowchart diagram for record selection.

Data for this review were sourced from three databases: Web of Science (WOS), ScienceDirect, and PubMed. The search query was: TS = (“music” OR “musical” OR “music therapy”) AND (“sleep” OR “sleep quality” OR “insomnia” OR “sleep disorders”). The search period was limited to 2019–2023, with the final search conducted on November 10, 2023. Based on this strategy, a total of 3,534 articles were identified. The specific screening process is illustrated in Figure 1. Ultimately, only 27 studies met the inclusion and exclusion criteria and were selected for further analysis (Figure 1).

## 3 Results

We extracted data on the types of sleep issues studied, sample size and characteristics, intervention designs, sleep measurement methods, and music materials, summarizing the main findings. The measurements and results are presented in Tables 2–4 according to the progression of this study’s content. In all these studies, a recurring topic is which type of MT is effective for specific types of sleep disorders. Therefore, this review also discusses the data from two perspectives: “Mechanisms of Music Therapy” and “Categories of Sleep Disorders Affected by Music Therapy.”

**Table 2:** EEG methods and effects of music therapy.

Author	Number	Sample	Independent variable	Electrodes	Equipment	Sleep measure	Music material	Findings
Liisa Kuula et al. (37)	20	Between the ages of 20–45, with a relatively stable sleep schedule	Slow breathing /Music listening	Six EEG channels at F3, F4, C3, C4, O3, O4, and contralateral mastoids (A1 and A2)	SOMNOmedics GmbH, Randersacker, Germany	PSQI, PSG	The first two tracks and the first part of the third track on Max Richter’s album “Sleep.”	Music listening may increase frontal beta1 power in SWS over the entire night.
Rebeca Sifuentes-Ortega et al. (36)	14	Healthy adults with good quality sleep (PSQI mean score 3.1 ± 1)	Within-subjects design	Ag-AgCl electrodes on scalp locations C3, C4, Cz, FCz	An Active-Two Biosemi System (Biosemi, The Netherlands)	PSG	Two non-isochronous rhythms, each with a duration of 2.4 s, continuously looped into sequences of 40.8 s.	The ability to process the auditory stream during sleep is limited by basic neurophysiological organization.
Gao et al. (38)	33	Participants with a habit of staying up late (PSQI scores 4–8)	Group 1 (*n* = 13), Group2 (*n* = 11), Control Group (*n* = 9)	F3, F4, C3, C4, O1, O2	An Alice 5 LDx system (Philips Respironics, PA, United States)	EEG, EOG, ECG, EMG	REM brain-wave music and SWS brain-wave music.	SWS brainwave music may positively affect sleep quality, whereas REM brainwave music or WN does not.

SWS, Slow-Wave Sleep; EEG, Electroencephalogram; EOG, Electrooculography; ECG, Electrocardiography; EMG, Electromyography; PSG, Polysomnography; PSQI, Pittsburgh Sleep Quality Index; PSG, Polysomnography; WN, White Noise; REM, Rapid Eye Movement.

**Table 3:** Music therapy effects by age group and music type.

Author	Number	Sample	Age	Independent variable	Sleep measure	Music material	Findings
Children and adolescents (0–18 years)							
Vito Giordano et al. (49)	64	Preterm infants born before 32 weeks of gestation	Approximately 30 weeks	Live music/Recorded music/Control	EEG	Soft whispers and humming; adding the melody of a child’s harp; Brahms’ lullaby	Music may help improve the quality of hospitalization for preterm infants.
Maksude Yildirim et al. (44)	50	Child with liver transplant	0–18	Therapeutic touch/Music rest	Actigraphy	A piece of music (such as a lullaby, or classical music) preferred by the patient or his/her family	Children in the music rest group had increased bedtime and total sleep time and shorter latency periods after undergoing surgery.
Sarah Bompard et al. (15)	12	Children	<12	Non-controlled pilot study	SDSC	The “Euterpe” method (uses a personalized soundtrack for each child)	Total SDSC scores improved after treatment, as well as sleep apnea disorders and sleep arousal transition disorders.
Sylka Uhlig et al. (50)	52	Eighth grade elementary school students	8–13	Experimental/Control	Actigraphy	Rap & Sing music	Rap&Sing MT had a moderate positive effect on sleep duration.
Youth (18–40 years)							
Lee et al. (23)	76	Stressed persons	19–65	ASMR/BB	PSQI	ASMR; BB	Sleep latency, total sleep time, time in bed were significantly improved in both group with sleep efficiency.
Minji Lee et al. (46)	15	Healthy right-handed subjects	Average age of 24.9 ± 1.81 years	Single group design	QEEG, ISI, PSQI	6 Hz BB; ASMR	Auditory stimulation induces the brain signals needed for sleep while putting the user in a state of psychological comfort.
Mehtap Kavurmaci et al. (4)	45	College students	CG = 21.44 ± 0.86 EG = 22.20 ± 1.32	Experimental/Control	PSQI	Hejaz	The mean PSQI scores of students in the experimental group measured after listening to music therapy were lower than the mean scores of students in the control group. Music therapy can improve students’ sleep quality.
Kamila Litwic-Kaminska et al. (17)	22	University athletes with decreased sleep quality	MEG = 22.36 MCG = 21.73	Experimental /Control	PSQI, Biofeedback device, Actigraphy, Daily Logs	Background music without suggestions (CG); recording with the relaxation training (EG)	AT seems to be an effective method for university athletes in improving subjective sleep quality, but further studies are necessary.
Ramachandran Krishna et al. (52)	30	cancer patients	>18	A single group pre-post research design	PSQI	Raga-Bilahari based music	Carnatic raga-Bilahari-based music intervention is effective among caregivers of cancer patients to reduce anxiety, sleep disturbances, somatic symptoms presentation, and their distress level.
Chenlu Gao et al. (45)	41	College students	18–33	TMR/Control	PSG, EEG	Distinctive classical music (Chopin, Beethoven, and Vivaldi)	TMR can affect sleep microarchitecture, specifically increased frontal theta activity averaged across SWS epochs throughout the night.

EEG, Electroencephalogram; aEEG, Amplitude-integrated Electroencephalography; ASMR, Autonomous Sensory Meridian Response; SDSC, The Sleep Disturbance Scale for Children; PSQI, Pittsburgh sleep quality index; PSG, Polysomnography; TMR, Targeted Memory Reactivation; MEG, Mean age of Experimental Group; MCG, Mean age of Control Group.

**Table 4:** Effects of music therapy on various sleep disorder categories.

Author	Number	Sample	Independent variable	Sleep measure	Music material	Findings
Benjamin Ho et al. (47)	10	Subjects with ISI scores ≥8	Single-Group	PSG	A combination of alpha and theta frequencies (4–12 Hz range), beginning with alpha (9–12 Hz), then theta (4–8 Hz), and then ending in alpha. The sound comprises the pulse tones and natural sounds.	A combination of pulse tone at alpha and theta frequencies may have a positive effect on people with mild insomnia.
Jespersen et al. (25)	57	Individuals between 18 and 65 years of age who meet DSM-5 criteria for insomnia disorder	Intervention/Active control/Waitlist control	PSQI, ISI, PSG, Actigraphy	The music genres were classical, jazz, new-age and ambient. All music was instrumental and characterized by a slow tempo (50–80 bpm), stable dynamics and a simple structure.	Bedtime music improves sleep quality and quality of life, but has no significant effect on insomnia severity.
Lee et al. (39)	43	Insomniacs between 20 and 59 years of age	Sham/Active	QEEG	6 Hz BB with Classical music, sounds of nature or pop songs.	Exposure to music with BB is likely to reduce the hyper-arousal state and contribute to sleep induction
Mei-Jou Lu et al. (40)	66	Patients with schizophrenia with sleep disorders.	Control/Intervention	PSQI	Soft music, country music, classical music, vocal music, popular songs in Mandarin or Taiwanese, and sounds of nature such as soft wind, bird song, or ocean sounds.	Music therapy demonstrated its merit on sleep disturbance among patients with schizophrenia.
Xian Wang et al. (24)	177	Asymptomatic patients with COVID-19 infection.	Treatment/Control	JSS	5-elements music.	Baduanjin Qigong and the five-elements music therapy may help to relieve anxiety and depression, improve sleep quality, and promote the perception of health in asymptomatic patients with COVID-19.
Haizhi Liu et al. (48)	91	Osteosarcoma patients	Control/Intervention	PSQI	Lyrical and natural background music/ rhythmic, cheerful, and lively music/fresh and elegant music/cheerful and hopeful music.	PSQI scores plummet.
Qi-Liang Zhang et al. (41)	222	Patients undergoing mechanical MVR	Music/Control	VSH	Included, but were not limited to, classical, folk, and pop music	Music therapy reduces early postoperative pain, relieves early postoperative anxiety, prolongs sleep duration, and improves sleep quality in patients after mechanical stroke.
KübraGÖ KALP et al. (57)	60	Older hematologic cancer patients	Control/Experimental	PSQI	Hejaz, Husseini and Neva compositions (nonverbal instrumental music) from the music and health series prepared by Tumata.	MT decreased PSQI and STAI mean scores of the experimental group. MT increases sleep quality and decreases the anxiety of older hematologic cancer patients.
Haoke Tang et al. (14)	100	Patients with small cell lung cancer scheduled to receive platinum-based chemotherapy	Control/Experimental	PSQI	(1) Individual music selection: preferred music (2) Collective music selection: the music with a simple structure, rhythm, and a cheerful melody.	MT promoted sleep quality of platinum-based SCLC patients.
Jeongmin Kim et al. (42)	133	Elderly patients in ICU	IMT/PL/Control	RCSQ	Air for G string, Allemande, Canon, Nocturne, and Swan.	Postoperative sleep quality was assessed using the RCSQ and was significantly better in the IMT group than in the control group.
Francesco Burrai et al. (43)	159	Heart failure patients	Control/Experimental	PSQI	A classical repertoire structured to avoid significant adrenergic stimulation and a raise of cortisol levels was chosen.	Listening to recorded classical music is a feasible, noninvasive, safe, and inexpensive intervention, able to improve QOL in patients with HF in the home-care setting.
Kabuk et al. (58)	36	Burn patients	Reflexology/Reflexology and Passive Music Therapy /Control	RCSQ	MP3 music recording of the Huseyni melody.	The quality of sleep on the fourth day was significantly higher in the experimental group than in the control group.
Pramita Dubey et al. (59)	15	Men between 18 and 40 years of age	Music/Non-music	PSG	432 Hz music.	Music at a frequency of 432 Hz had a relaxing effect on the brain’s electroencephalographic activity during daytime napping, especially for individuals with prolonged latencies
Kusumandari et al. (60)	10	No specific requirements (men = 5 women = 5)	Intervention group (before and after intervention)	EEG	Sundanese music.	With Sundance music, brain activity becomes calm and relaxed, unlike before the treatment.

ESD, Electronic Sleep Diary; PSG, Polysomnography; PSQI, Pittsburgh sleep quality index; EEG, Electroencephalogram; QEEG, Quantitative Electroencephalography; PSAS, Pre-Sleep Arousal Scale; DSISD, Duke Structured Interview for Sleep Disorders; ISI, Insomnia Severity Index; ESS, Epworth Sleepiness Scale; JSS, The Jenkins Sleep Scale; VSH, Veran and Snyder Halpern Sleep Scale; RCSQ, The Richards-Campbell Sleep Questionnaire.

### 3.1 Effectiveness of music therapy

**Figure 2:**
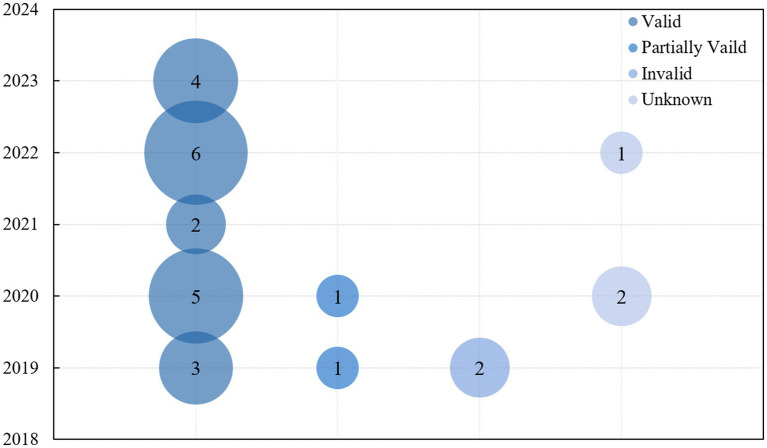
Analysis of research validity.

We first focused on the effectiveness of music therapy. To provide a clear visualization of the data from the 27 studies included in this review, intervention-related metrics were integrated and presented through graphical representations. Figure 2 illustrates the distribution of the 27 studies by publication year and their reported effectiveness. Specifically, the studies were categorized into three groups: 20 were identified as effective, 2 as partially effective, and 5 as either ineffective or inconclusive. The bubble chart (Figure 2) uses four distinct colors to represent these categories, while the vertical axis corresponds to the publication year. The size of each bubble reflects the number of studies published in that particular year.

Notably, studies categorized as “effective” accounted for the majority, comprising 74% of the total, while “partially effective” and “ineffective/inconclusive” studies were fewer in number. This distribution underscores the widespread recognition of the therapeutic benefits of music therapy. Over time, the proportion of studies reporting positive outcomes has steadily increased, particularly since 2019, indicating a robust and ongoing validation of music therapy’s efficacy.

### 3.2 Mechanisms of music therapy

We first focused on the methods used in current research developments. Overall, the influence of music therapy on sleep is primarily characterized through objective sleep monitoring (physiological aspect) and subjective sleep measurement (psychological aspect). Additionally, understanding effective music materials and target populations is essential for a comprehensive grasp of current methodologies.

#### 3.2.1 Objective sleep monitoring

Among the 27 empirical studies included in this review, 15 utilized objective sleep monitoring, employing methods such as EEG [6 studies, including 2 with Quantitative Electroencephalography (QEEG)], polysomnography (5 studies), and actigraphy (4 studies). Detailed indicators for these studies are shown in Table 2. Passive music listening’s effect on brain waves during sleep is particularly noteworthy. Sifuentes-Ortega et al. (36) focused on EEG responses across different sleep stages, analyzing music’s modulation effect on brainwave frequencies. Their results demonstrated that in the wakeful state, music induced marked EEG frequency responses to different rhythms; this effect was retained during REM sleep but disappeared in NREM sleep (36). Similarly, Kuula et al. (37) reaffirmed the significance of brainwave representation in a randomized controlled trial investigating the overall impact of music and slow pre-sleep breathing on sleep quality. The results showed that music increased the percentage of stage N3 throughout the night, significantly correlating with the power spectral density in specific EEG bands, suggesting that music may improve sleep structure by increasing the proportion of deep sleep (37).

Notably, the application of brainwave monitoring extends beyond the objective representation of sleep states. Brainwave music, generated by integrating brainwave frequencies with music, produced positive effects in a study involving subjects with frequent late nights. This intervention successfully reduced EEG power spectral density in the Delta wave band and shortened sleep onset latency, thereby improving sleep quality, whereas the control group (REM brainwave music and white noise) did not exhibit similar effects (38).

#### 3.2.2 Subjective sleep measurement

Among the 27 studies included in this review, 16 conducted subjective sleep measurement using various scales. The Pittsburgh Sleep Quality Index (PSQI) was used in 12 studies, the Richards-Campbell Sleep Questionnaire (RCSQ) in 2 studies, the Insomnia Severity Index (ISI) in 1 study, the Sleep Disturbance Scale for Children (SDSC) in 1 study, the Verra and Snyder-Halpern Sleep Scale (VSH) in 1 study, the Jenkins Sleep Scale (JSS) in 1 study, and sleep diaries in 1 study (25). Additionally, eight studies evaluated participants’ anxiety levels (14, 23, 24, 39–43), while five studies focused on patients’ depressive symptoms (23, 24, 39, 43, 44).

**Figure 3:**
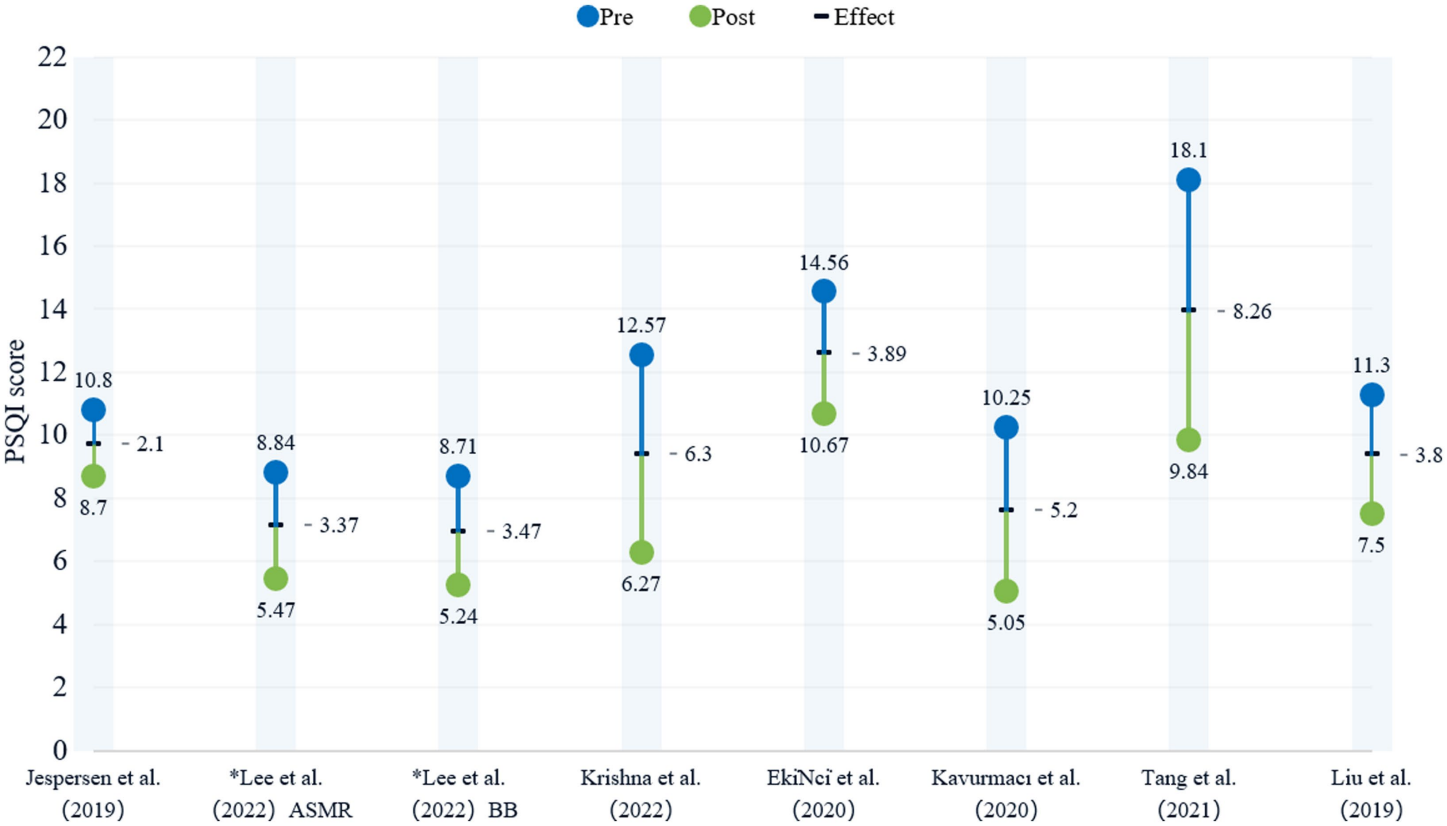
Pittsburgh sleep quality index (PSQI) scores and pre-post differences.

Given that the PSQI was the most commonly used tool in these studies, it is discussed separately. However, only 8 groups from 7 studies provided complete pre-and post-intervention PSQI data. To illustrate this, a bead plot was created, where * indicates two experimental groups within the same study. Blue and green represent the PSQI scores before and after the intervention, respectively, with the specific differences in scores marked in black. As shown in Figure 3, PSQI scores significantly decreased following music therapy, with an average reduction of 4.55. Three studies reported a reduction greater than this average. Notably, participants with higher baseline PSQI scores, such as cancer patients, showed more substantial improvements in sleep quality compared to those with lower baseline scores.

#### 3.2.3 Categories of effective music therapy materials

MT positively impacts sleep by regulating individuals’ emotions and cognitive states. Among the 27 studies, classical music was the most frequently used material (25, 39–45), followed by binaural auditory beats (BB) (23, 39, 46), Autonomous Sensory Meridian Response (ASMR) sounds (23, 46), and natural sounds (47, 48). From the perspective of musical characteristics, most studies opted for slower instrumental or pure music, rather than songs with lyrics or fast-tempo tracks.

#### 3.2.4 Target populations

To present the effects of MT on different age groups clearly, participants were categorized into four groups: children and adolescents (0–18 years), young adults (18–40 years), and middle-aged and older adults (over 40 years). Table 3 documents sample size, participant demographics, age range, independent variables, sleep monitoring methods, music materials, and results for each age group.

##### 3.2.4.1 Children and adolescents (0–18 years)

The youngest population successfully impacted by MT in this review was preterm infants. Preterm infants typically require care in the Neonatal Intensive Care Unit (NICU), where the environment may adversely affect their sleep (49). Giordano et al. (49) randomly assigned 64 preterm infants to a live music group, a recorded MT group, and a control group. Amplitude-integrated Electroencephalography (aEEG) was used as the primary assessment measure to monitor the Quiet Sleep (QS) stage. In the live music group, a certified music therapist used touch, vocal interaction, and a small harp, while the recorded music group listened to “Brahms’ Lullaby.” Results showed significant improvements in both music groups during the first and second quiet sleep stages, whereas no changes were observed in the control group (49).

The sleep quality of slightly older children also benefited from MT. Children (0–18 years) who had undergone liver transplants were divided into a therapeutic touch group (*N* = 25) and a music relaxation group (*N* = 25) for three consecutive days of therapeutic touch and music relaxation sessions (20 min each). Sleep was assessed before and after each session using actigraphy. Results indicated that post-operative bed time, total sleep time, and sleep efficiency improved in the therapeutic touch group, while the music relaxation group showed increases in bed time and total sleep time, highlighting the positive effects of both interventions on sleep in liver transplant children (44).

Additionally, Bompard et al. (15) utilized an innovative “Euterpe” music therapy method (incorporating sound and various types of sensory stimuli) for children with developmental disabilities. This personalized music creation combined the child’s vocal memories with family scenes, maternal voice, and other familiar sounds. After 12 days of treatment, subjective assessments of sleep quality were made using the Sleep Disturbance Scale for Children (SDSC). Results indicated significant improvement in sleep quality among children with developmental delays (15).

Adolescents also experienced positive effects. A group of adolescents (*N* = 52) participated in weekly rap MT sessions (45 min each) over 4 months, conducted by a music therapist. Sleep variables were objectively assessed using actigraphy. Results showed a general reduction in total sleep time across participants, though rap MT did not significantly improve overall sleep quality. However, it did have a modest positive impact on sleep duration (50).

##### 3.2.4.2 Young adults (18–40 years)

Among young adults, university students were the primary focus in recent studies. Litwic-Kaminska et al. (17) conducted music therapy sessions for college athletes during pre-sleep training, involving 31 student athletes with PSQI scores ≥5. The experimental group listened to a self-produced recording based on Autogenic Training (AT) to aid relaxation, while the control group listened to background music. During the sessions, pulse, heart rate, skin conductance, and temperature were recorded using four sensors on the fingers, and breathing was measured via an abdominal sensor. Both objective (sensors) and subjective (PSQI) assessments indicated that overall sleep quality significantly improved in the experimental group, with no notable change in the control group (17).

Lee et al. (23, 46) conducted two interrelated studies within 5 years to verify the effectiveness of ASMR and BB as MT materials. The first study involved 76 stressed adults, with sleep quality subjectively assessed using PSQI and ISI. Results showed that both ASMR and BB significantly improved sleep quality (46). The second study involved 15 healthy adult males, using EEG to monitor sleep metrics. This study combined ASMR and BB as “Combined Stimuli” (CS) for sleep induction. Results indicated that CS induced brain activity at 6 Hz, within the theta frequency range, associated with stage 1 of non-REM sleep (NREM) (23).

Music has also shown promise in promoting sleep while activating memory. A study with 50 undergraduate students used polysomnography (PSG) for objective sleep monitoring. The Targeted Memory Reactivation (TMR) group listened to classical music pieces by Chopin, Beethoven, and Vivaldi, while the control group listened to white noise before sleep and during slow-wave sleep. Results revealed that the TMR group experienced increased theta activity in the frontal cortex during slow-wave sleep, indicating that TMR influenced microstructural sleep characteristics, especially enhancing average frontal theta activity throughout the night (45). Additionally, studies confirm that the Hejaz melody exerts a soothing and calming effect throughout the night, improving sleep quality in university students (4). Lastly, Snipes et al. (51) introduced supplementary methods (such as slow breathing exercises) to enhance MT. EEG data analysis showed that the combined use of these methods increased the likelihood of deep sleep, enhancing delta power during the Slow Wave Sleep (SWS) stage, thereby promoting higher quality deep sleep (51).

##### 3.2.4.3 Middle-aged and older adults (>40 years)

Krishna et al. (52) conducted a novel study with 30 cancer caregivers, including 20 middle-aged and older caregivers, using Bilahari Raga from Carnatic music as the therapeutic material. This month-long MT intervention demonstrated significant effects in reducing caregivers’ anxiety and improving sleep quality (52). It is noteworthy that in the empirical studies included in this review, sleep disorders in middle-aged and older adults commonly co-occur with pain and anxiety stemming from various health conditions, which will be discussed in detail in section 4.2.3.

### 3.3 Categories of sleep disorder effected by music therapy

Previous studies have identified several key areas within sleep-related issues and provided valuable insights into the efficacy of MT. However, consensus on defining categories of insomnia remains elusive. The International Classification of Sleep Disorders (Third Edition, ICSD-3) includes insomnia disorders such as chronic insomnia, short-term insomnia, and other insomnia types (53), and this classification standard has been widely recognized in the medical field as authoritative. In the included studies, however, insomnia categories are primarily determined by referencing established scales or multiple physiological indicators (e.g., polysomnography, ISI, or PSQI results). Furthermore, this categorization is complicated by the coexistence of various comorbidities. To better illustrate the results, we reclassified the studies based on ICSD-3 standards (Table 4 presents sample size, participant demographics, independent variables, sleep monitoring methods, music materials, and outcomes). Specifically, in the 27 studies included in this review, categories of sleep disorder effected by MT were as follows:

1. Chronic Insomnia (1 study included): ICSD-3 defines chronic insomnia as having symptoms for at least 3 months, occurring at least 3 times per week (53). Participants with insomnia duration meeting this criterion were classified accordingly.
2. Mild Insomnia (2 studies included): Participants with ISI scores between 8 and 15 exhibited only mild insomnia symptoms and did not meet the diagnostic criteria for insomnia disorder per international standards, thus classified as mild insomnia.
3. Other Insomnia (14 studies included): Studies addressing sleep issues arising from comorbid conditions but without a specific definition of insomnia symptoms were grouped under other insomnia.
4. Non-Pathological Health Concerns (10 studies included): Studies focused on enhancing sleep quality in healthy individuals were classified within this category.

#### 3.3.1 Chronic insomnia

Jespersen et al. (25) conducted the only recent study specifically addressing chronic insomnia. They performed a three-week randomized controlled trial involving 57 patients with chronic insomnia (average insomnia duration of 10.3 years). Participants were randomly assigned to a music group, an audiobook control group, and a waitlist control group. Objective sleep indicators were assessed using polysomnography and actigraphy. Subjective sleep measurements (including ISI and PSQI) revealed that the music group experienced a significant reduction in insomnia severity and an improvement in quality of life compared to the control groups. However, objective sleep measurements indicated no significant differences in sleep parameters across groups, and further analysis showed no impact of music on the amount of time spent in different sleep stages. Although music had a notable effect on sleep perception and quality of life in chronic insomnia patients, there was no significant impact on objective sleep metrics or insomnia severity (25).

#### 3.3.2 Mild insomnia

Similar to chronic insomnia, research attention on mild insomnia remains limited. Lee et al. (39) conducted a trial using music embedded with binaural beats (BB) for subclinical insomnia patients with ISI scores below 15. The results indicated an increase in *δ* and *θ* power and a decrease in *α* power during exposure to BB-enhanced music, suggesting that music listening promoted relaxation and sleep. The increase in θ waves also indicated that BB might induce sleepiness (39). Additionally, Ho et al. (47) employed an innovative intervention for mild insomnia by using an audio pillow with embedded speakers to deliver brainwave entrainment sounds (α and θ beats) that synchronized with brain activity. This technology allowed for observation of sleep stage proportions. Results showed that participants using the audio pillow experienced a slight reduction in time spent in stages 1 and 2, with an increase in time spent in stage 3 and REM sleep. Although these changes were not significantly different from the control group, the audio pillow group showed a significantly shorter sleep onset latency, indicating a positive effect on falling asleep. Furthermore, there was no significant change in melatonin levels before and after the experiment, suggesting that light, rather than sound, might be the primary influence on melatonin production during sleep induced by the audio pillow (47).

#### 3.3.3 Other insomnia disorders

Insomnia commonly coexists with various physiological and psychological disorders, accounting for 51.85% of the studies reviewed.

##### 3.3.3.1 Sleep structure in patients with schizophrenia

Schizophrenia patients often experience abnormal sleep structures, such as deficits in SWS and reduced REM sleep duration (54, 55). Lu et al. (40) conducted a MT intervention for patients with chronic schizophrenia experiencing sleep disturbances. The severity of sleep disturbances was evaluated using the PSQI, with patients scoring above 5 included in the study. Patients attended MT sessions in the activity room from 9:00 to 10:00 PM over 4 weeks. Results indicated a significant reduction in PSQI scores in the MT group, though the effect on sleep quality improvement was less pronounced for older patients (40).

##### 3.3.3.2 Sleep issues in asymptomatic COVID-19 patients

Asymptomatic COVID-19 patients often experience insomnia due to prolonged negative emotions, viral infection, and subsequent isolation, which can lead to sleep disturbances, anxiety, and depression. Wang et al. (24) conducted a study involving 200 asymptomatic COVID-19 patients, where the treatment group practiced Baduanjin Qigong (i.e., 八段锦气功) and received Five Elements music therapy. Specific music pieces were chosen based on the effects of different modes on internal organ functions, such as using the “Yu” (i.e., 羽) tone to nourish kidney yin, “Shang”(i.e., 商) tone to moisten the lungs, and “Zhi” (i.e., 徵) tone to clear heart fire. These musical pieces were played at designated times to aid in adjusting patients’ physical and emotional states. The results showed that the combined intervention significantly improved sleep quality compared to patients who received only standard care (24).

##### 3.3.3.3 Sleep issues in medical settings

Patients in medical settings often experience poor sleep quality due to hospital noise, lighting, temperature, and care interruptions (56), which can contribute to insomnia. This issue has received significant attention in the literature.

Liu et al. (48) conducted a controlled study on osteosarcoma patients, where the MT group received a combination of mindfulness-based stress reduction (MBSR) and diverse music selections, including calming natural background music, rhythmic upbeat music, and soothing hopeful tunes. The control group received standard care only. Each session began with 30 min of MT by a professional music therapist, followed by MBSR led by a psychologist, and concluded with another 30 min of music. Pain (Wong-Baker Faces Pain Rating Scale, WBRS), anxiety (Hamilton Anxiety Rating Scale, HAM-A), and sleep quality (PSQI) were assessed before and after treatment. Results indicated that MT significantly reduced pain and anxiety levels and improved sleep quality in osteosarcoma patients (48). Similarly, a study involving hematologic cancer patients divided participants into control (*n* = 30) and experimental groups (*n* = 30). Before the intervention, both groups had poor sleep quality with average PSQI scores of 14.50 ± 2.70 (control) and 14.56 ± 2.00 (experimental). The experimental group received MT (30–40 min each night for 1 week), while the control group did not receive MT. Results showed a significant reduction in PSQI and State–Trait Anxiety Inventory (STAI) scores in the MT group, indicating that MT effectively improved sleep quality and reduced anxiety in elderly cancer patients (57).

Six-step music therapy was also shown to alleviate pain, reduce anxiety, and improve sleep quality in patients undergoing platinum-based chemotherapy for lung cancer. The study included 100 small-cell lung cancer patients randomly assigned to a MT group or a control group. Music selections were chosen based on patient preferences, and the six steps included: (1) improvisation (group MT); (2) music-assisted progressive muscle relaxation (individual pre-sleep therapy); (3) music imagery (individual therapy during chemotherapy); (4) music breathing exercises (group therapy after each chemotherapy injection); (5) instrument playing (group therapy during chemotherapy); and (6) rhythmic activities (group therapy). Patients completed the Self-Rating Anxiety Scale (SAS), Visual Analog Score (VAS), and PSQI before chemotherapy, the first day post-chemotherapy, and 5 days post-chemotherapy. The MT group showed significantly reduced anxiety and pain scores, with higher PSQI scores than the control group (14).

Interestingly, Kim et al. (42) demonstrated the efficacy of a single MT session for post-operative elderly patients in the ICU. A total of 133 patients were randomly assigned to an Interactive Music Therapy (IMT) group, a Passive Listening (PL) group, and a control group. The IMT group received a 20-min interactive music session led by a music therapist, followed by individual evening music listening, while the PL group received only evening music listening, and the control group received standard care without MT. Melatonin and cortisol levels were measured before surgery, on the day of surgery, and the day after. Sleep quality was assessed using the RCSQ. Results indicated an increase in melatonin levels in the IMT group, while cortisol levels showed no significant difference between groups. Interactive music was found to significantly improve subjective sleep quality in ICU post-operative elderly patients (42).

Moreover, the positive effects of MT have been repeatedly validated in other patient groups, including those undergoing Mitral Valve Replacement (MVR) surgery (41), heart failure patients (43), and burn patients (58). After 30-min music therapy sessions, early post-operative MVR patients experienced significant reductions in pain (VAS) and anxiety (SAS), with Verran and Snyder-Halpern (VSH) results indicating improved sleep duration and subjective sleep quality scores compared to the control group (41). Heart failure patients who listened to music for 30 min daily for 3 months reported significantly improved subjective sleep quality (assessed by PSQI) compared to the control group, suggesting MT’s efficacy in improving sleep for heart failure patients (43). In a 4-day randomized controlled trial with burn patients, the passive MT group (listening to an mp3 recording of the Huseyni melody played by the OTAG Music Center) reported significantly better sleep quality (measured by RCSQ) and reductions in pain and anxiety compared to the reflexology massage group and control group (58).

#### 3.3.4 Non-pathological health concerns

Sleep issues among healthy individuals also warrant attention. Pramita et al. (59) conducted a study on 15 healthy males with a history of delayed sleep onset to assess the impact of specific frequency music on sleep structure. Participants underwent sleep studies over a week with and without music intervention, measuring parameters such as EEG, Electrocardiogram (ECG), and Electromyography (EMG). Results indicated that the music group exhibited increased alpha power during sleep compared to the non-music group. Music at a frequency of 432 Hz had a significant relaxing effect on the brain, though its impact on sleep onset latency remained unclear, particularly among individuals with delayed sleep onset (59). Another study used EEG to objectively monitor sleep and found that Sundanese music from West Java, Indonesia, promoted calm and relaxation in brain activity, positively contributing to daytime sleep quality (60).

## 4 Discussion

This study reviews empirical research from the past 5 years on the effects of music therapy (MT) on sleep, offering new theoretical support for future applications and research in MT. We included 27 recent empirical studies to examine effective categories and mechanisms of MT. The analysis revealed that MT significantly improved subjective sleep quality, though its effects on objective sleep metrics varied, suggesting that music primarily influences sleep quality through subjective pathways, such as emotional regulation and anxiety relief. In terms of effectiveness, the majority of studies (74%) showed positive results, with music therapy’s effectiveness being increasingly validated in recent years. Unlike previous meta-analyses that focused mainly on randomized controlled trials, this review included a variety of experimental paradigms, extending the evaluation dimensions to provide a comprehensive assessment of MT ‘s effects.

### 4.1 Gaps in the methodology

#### 4.1.1 Objective sleep monitoring

There is no perfect sleep assessment method, but objective evaluation remains crucial for accuracy (61). Combining objective and subjective approaches can enhance assessment reliability. Music therapy’s effects on physiological metrics are often represented through measures like sleep onset latency, total sleep duration, and specific sleep stages (e.g., deep sleep) (52, 62). It has been demonstrated that music significantly shortens sleep onset time and extends the duration of NREM sleep (37). Furthermore, the use of various physiological signal monitoring techniques has enriched our understanding of the physiological mechanisms of MT. Music intervention may aid in reducing cortical arousal levels, helping individuals to enter stable sleep more rapidly. Insomnia patients typically exhibit heightened cortical activity before and during NREM sleep, which can hinder natural sleep onset (63). Despite its value, objective sleep monitoring has not received adequate attention. Future studies should further apply objective tools like EEG to monitor the impact of different types of music on brain activity in insomnia patients, providing quantitative data to support a theoretical framework for music intervention mechanisms (49).

#### 4.1.2 Subjective sleep measurement

Subjective sleep measurement methods are well-established, with many validated tools available. Instruments such as the PSQI, RCSQ, and ISI are widely used to assess individuals’ personal sleep experiences (64). PSQI, due to its universal applicability, reliability, and validity, is the most commonly used subjective sleep assessment tool. Interestingly, reported sleep quality often correlates with various emotional factors. MT has been shown to positively affect sleep quality, as evidenced by an average reduction of 4.55 in PSQI scores. Notably, greater improvements were observed in studies exceeding this average reduction, as well as among participants with higher baseline PSQI scores, such as cancer patients, as demonstrated in Tang et al. (14). The positive effect of MT on sleep quality is also linked with improvements in quality of life (50, 65), relief from anxiety and depression (57), and enhanced life satisfaction (18). Combining subjective sleep measurement with psychological assessment tools offers an excellent perspective for investigating the psychological mechanisms of MT’s impact on sleep. Future research could integrate subjective sleep quality and emotional assessment tools to further verify MT‘s positive impact on overall health.

#### 4.1.3 Categories of effective music therapy materials

MT encompasses a diverse array of therapeutic approaches, emphasizing personalization and context-specific application. Different music materials used during MT can have varied impacts on sleep quality, making it essential to explore the most effective therapeutic approaches. The most commonly used music materials in the reviewed studies were classical music, natural sounds, and 432 Hz audio. Music materials typically share features such as a slow tempo, minimal rhythm changes, and moderate pitch variation (66). Tempo synchronized with the average heart rate (60–80 bpm) is an important indicator, aiding relaxation (67, 68). Additionally, emerging audio technologies (e.g., BB, audio entrainment, and brainwave music) have shown promising results in improving mild insomnia. MT interventions are generally set to play music 30 min to an hour before sleep, with a duration of 20–30 min, to help individuals reach a relaxed state conducive to sleep onset without disrupting sleep structure (15, 20). The music volume during MT should be controlled at 40–50 decibels to avoid disturbing natural sleep (20).

Many researchers also prefer using participants’ preferred music to improve relaxation and reduce negative emotions, which can facilitate sleep. However, such music may induce Involuntary Musical Imagery (INMI, or “Earworm”), due to familiarity, melody characteristics, and lyrical content differences (69). INMI is the spontaneous recollection and repetition of a melody in one’s mind (69), a phenomenon common in daily life (70). Studies suggest that INMI could lead to continuous “replaying” of melodies in the mind, potentially disrupting relaxation and negatively impacting sleep (71, 72). Additionally, specific groups, such as musicians and individuals with obsessive-compulsive disorder (70), may experience INMI symptoms more frequently. This raise concerns that familiar, preferred music might bias sleep intervention outcomes. However, no recent studies have addressed this issue. Avoiding familiar or popular music in favor of playlists curated by professional music therapists could potentially reduce INMI’s interference, a hypothesis that should be tested in clinical studies to clarify INMI’s actual impact in MT.

#### 4.1.4 Target populations

Existing research suggests that m MT has varied effects on sleep improvement across different age groups. Research on middle-aged and older adults is more common, focusing primarily on music’s role in addressing age-related sleep issues (e.g., insomnia, reduced sleep quality) (40). These studies typically demonstrate that MT improves sleep quality in middle-aged and older adults by reducing anxiety and alleviating mood fluctuations, but such improvements may not be fully applicable to younger populations (57). For example, a study on cancer caregivers found that music in specific modes effectively reduced anxiety and improved sleep quality, though the same effect might not apply to other healthy adults (52).

In comparison, studies on MT for sleep among children, adolescents, and young adults are limited. Existing research indicates that certain groups, such as preterm infants (49) and children with developmental delays (15), may be more responsive to music’s positive effects. Adolescents, as a unique group, have complex sleep intervention needs, and MT has shown limited effects on their overall sleep quality, possibly only improving specific sleep variables (e.g., sleep duration) (50). This effect has been shown to occur indirectly through relaxation and anxiety relief (17). In healthy young adults without sleep disorders, music’s impact on deep sleep enhancement appears limited (37).

In summary, while MT demonstrates certain positive effects on sleep across different age groups, current research on children, adolescents, and young adults remains insufficient. Future studies should address the specific needs of these age groups to develop more targeted MT strategies, optimizing the application of MT across diverse age demographics.

### 4.2 Gaps in the research

Insomnia is one of the most common health issues and the most prevalent sleep disorder among the general population (73). While the effectiveness of traditional treatments, such as medication and cognitive behavioral therapy, has been established, only a small percentage of patients pursue these approaches, largely because they are predominantly accessible in academic and research settings (74). Against this background, MT presents considerable potential as a convenient and flexible non-pharmacological intervention (75).

#### 4.2.1 Potential bias in subjective and objective insomnia evaluation standards

A primary gap in current research is the inconsistency in evaluation standards. Insomnia diagnosis relies mostly on subjective assessments of sleep insufficiency, yet discrepancies often arise between subjective complaints and objective measurements. Studies on music’s effects on objective sleep metrics are limited, and results show variability compared to subjective sleep quality (16). This variability may result from factors like the protective role of positive emotions (63), heightened cortical arousal in insomniacs (76), and limitations in assessment methods.

Music improves sleep quality by modulating brain activity. It reduces beta waves linked to tension, enhances alpha waves associated with relaxation, and promotes parasympathetic activity, lowering cortical arousal and facilitating relaxation (77, 78). However, insomniacs’ heightened sensitivity to stimuli during sleep often prevents deep sleep, reducing MT’s effectiveness on objective measures (63). These findings are consistent with Chang et al. (19). Some studies suggest music improves both subjective and objective sleep, as shown by reduced N1 duration. However, this evidence comes from nap studies and excludes rapid eye movement (REM) sleep, leaving its relevance to general sleep disorders unclear (16).

Music also improves subjective sleep perception through emotional regulation. It activates the auditory cortex and limbic system (e.g., the amygdala), reduces anxiety, and induces dopamine release, enhancing positive emotions and subjective sleep evaluations (79, 80). Yet, these subjective improvements do not always align with physiological changes. Insomniacs often experience sleep illusion, perceiving wakefulness despite EEG data indicating light sleep (81, 82).

Limitations in assessment tools worsen the gap between subjective and objective evaluations. Polysomnography (PSG), while effective at capturing physiological states, fails to represent the restorative experience central to sleep perception (80–82). These limitations contribute to inconsistent findings and hinder a full evaluation of MT’s effects. Combining subjective and objective methods is essential for bridging this gap and better understanding MT’s impact.

#### 4.2.2 Variability in classification standards leading to imbalanced focus on different sleep problems

This review’s investigation into effective intervention categories was limited by the inconsistency in sleep disorder definitions across included studies. Researchers seem to employ varied standards, such as ICSD-3, ISI, and PSQI, resulting in insufficient focus on specific sleep disorders, making it challenging to cluster studies into a cohesive theoretical framework. Inconsistent standards may also lead to insufficient inclusion of certain conditions (83), restricting comprehensive attention to sleep disorders. For instance, chronic insomnia places a heavy burden on individuals and society, manifesting in reduced quality of life, increased absenteeism, and decreased work productivity (84). Moreover, chronic insomnia is often comorbid with physical and mental disorders (73, 85, 86). However, relying solely on insomnia duration as a criterion poses theoretical limitations and lengthens the experimental cycle, limiting the scope of investigation (25). Similarly, while mild insomnia lacks the prominent features of sleep disorders, it can develop into more severe sleep issues if not addressed early (87, 88). Therefore, effective intervention for subclinical insomnia is key to preventing the progression of insomnia. Since 2019, only two studies have used ISI scores (ISI = 8–15) to define mild insomnia when selecting participants. This metric is feasible but not fully aligned with international sleep disorder standards. Objective sleep monitoring results in these two studies showed positive effects of music, including reduced sleep onset latency (47) and increased theta wave power (39), confirming MT’s efficacy for mild insomnia patients. Nonetheless, the possibility of bias cannot be ruled out. To overcome this limitation, future studies should adopt standardized and replicable diagnostic criteria to ensure consistency and comparability in research outcomes.

#### 4.2.3 Difficulty in controlling variables due to comorbidity

Emotions and pain significantly impact sleep quality, with insomnia and poor sleep quality often exacerbating negative emotions, such as anxiety and depression, while high sleep quality is associated with positive emotions (63). Moreover, there is a bidirectional relationship between anxiety and future insomnia, as insomnia can further exacerbate future anxiety (89). Multiple studies confirm MT’s effectiveness in promoting sleep and managing pain and anxiety (14, 24, 41–43, 48, 57, 58). In this review, 25% of the studies involved participants with comorbid conditions, where sleep disturbances were often accompanied by one or more disorders, such as schizophrenia, COVID-19, osteosarcoma, hematologic cancer, lung cancer, heart failure, and burns. These conditions often trigger persistent anxiety and pain, further impacting sleep quality. Postoperative patients, for instance, are prone to insomnia due to physical trauma and pain during recovery. Pain not only causes physical discomfort but also induces negative emotions, such as anxiety, which in turn disrupt sleep. This dual effect underscores the importance of pain management in postoperative recovery. Effective pain control can reduce anxiety and significantly improve sleep quality, possibly due to the protective value of positive emotions induced by music, particularly for subjective sleep perception (63). For postoperative patients, hospital noise, especially at night, can severely disturb sleep. Reducing noise levels in hospital environments can effectively alleviate sleep disturbances in hospitalized cancer patients (90). Although the positive outcomes have been repeatedly validated, these symptoms differ from the symptoms of insomnia as defined by authoritative medical standards, and their causes are influenced by diverse and individual factors. Sleep studies on patients with multiple comorbidities face challenges in variable control, highlighting the need for refined research designs and control strategies in future studies, particularly to determine how to isolate the effects of comorbidities on sleep during interventions to enhance the scientific rigor and reliability of research results.

#### 4.2.4 Mechanisms and efficacy in non-pathological health conditions require further exploration

Some studies in this review specifically focused on healthy individuals, exploring the sleep-promoting effects of music under non-pathological conditions. Results indicated that music positively influenced subjective sleep experience, reduced sleep onset latency, and improved daytime nap quality in healthy individuals (59, 60). Although MT has been widely applied among populations with sleep disorders, its mechanisms and efficacy in healthy individuals without sleep disorders remain underexplored (15, 16). Emotional regulation through music may be a key pathway for enhancing sleep quality in healthy individuals (16, 63). Stress and negative emotions from daily life can influence sleep onset speed and depth (60, 63). Music can effectively reduce sympathetic nervous system activity, helping listeners enter a relaxed state (17, 37). In studies with healthy populations, specific types of music have been shown to promote higher quality sleep by modulating brainwave activity (17, 60). When healthy individuals listen to calming music before bed (e.g., slow-tempo instrumental music) (4, 37), Sundanese music (60), or music with a frequency of 432 Hz (4, 17, 59), they commonly report shorter sleep onset latency, prolonged deep sleep stages, and higher subjective sleep quality. However, current research mainly focuses on music materials as variables in discussing improvements in sleep quality among non-pathological populations. Future studies should consider other influencing factors, such as psychological state, lifestyle, and environmental factors, to comprehensively reveal the mechanisms and long-term efficacy of non-pathological health interventions.

## 5 Conclusion

This study reviewed empirical research from the past 5 years on music therapy’s effects on sleep, providing theoretical support and directional recommendations for the application and development of music therapy. By analyzing 27 studies with different experimental paradigms, we found that music therapy significantly enhances subjective sleep quality, particularly through subjective perception pathways like emotional regulation and anxiety relief. However, the effects of music therapy on objective sleep metrics were inconsistent, suggesting that the mechanisms through which music improves sleep are complex and may involve substantial individual differences. Unlike traditional meta-analyses, this review expanded the evaluation dimensions and comprehensively assessed various research findings, offering a broader perspective on the complex mechanisms underlying music therapy.

Despite its promising potential for promoting sleep, future research on music therapy still faces several directional challenges and opportunities. First, in terms of sleep monitoring, it is essential to integrate subjective and objective sleep assessment methods to jointly reveal the physiological and psychological effects of music therapy. Additionally, the current selection of intervention materials for music therapy remains relatively limited; future studies should further explore more precise and personalized music materials to maximize therapeutic efficacy and reduce unnecessary adverse effects, such as the potential interference from Involuntary Musical Imagery (INMI). Furthermore, research on different age groups and populations (e.g., children, adolescents, healthy individuals, and patients with multiple comorbidities) is limited. Expanding studies to include these participant groups could help develop tailored music therapy programs that meet diverse needs.

In conclusion, the multiple effects of music therapy on improving sleep quality are preliminarily established, but further exploration of its mechanisms and applicability is necessary through more standardized, rigorous experimental designs and diversified evaluation dimensions. Future research should incorporate various intervention variables, including music materials, psychological state, lifestyle, and environmental factors, to provide more scientifically robust empirical support for the broader application of music therapy in sleep health.

## 6 Limitation

This review has several limitations that may affect the general applicability of its findings. On one hand, regarding literature selection, this study included only empirical research from the past 5 years. Although this time limitation ensures the timeliness of the included studies, it also results in a relatively small number of overall studies. While the selected studies cover a diverse range of participant groups, the limited number of studies may not fully represent the effects of music therapy on patients with various types of sleep disorders. Future research could consider expanding the timeframe to gain a more comprehensive perspective.

On the other hand, the ambiguous definitions of sleep disorders in the included studies, along with differences in research design and methodology, may also affect the integration of results. The types of music used, duration of trials, and evaluation metrics varied across studies, complicating the overall assessment of effectiveness. As theory progresses, future research could address this limitation by providing diverse insights on these aspects.
